# Environmental Impact of Xenobiotic Aromatic Compounds and Their Biodegradation Potential in *Comamonas testosteroni*

**DOI:** 10.3390/ijms252413317

**Published:** 2024-12-12

**Authors:** Yapeng Li, Huixin Fan, Boqiao Li, Xiaobo Liu

**Affiliations:** 1 School of Environmental and Biological Engineering, Nanjing University of Science and Technology, 200 Xiaolingwei Street, Nanjing 210094, China; 918102270123@njust.edu.cn (Y.L.); boqiaoli@njust.edu.cn (B.L.); 2 Key Laboratory of Metabolic Engineering and Biosynthesis Technology, Ministry of Industry and Information Technology, Nanjing University of Science and Technology, 200 Xiaolingwei Street, Nanjing 210094, China

**Keywords:** xenobiotic aromatic compounds, environmental impact, biodegradation, bioremediation, *Comamonas testosteroni*

## Abstract

Xenobiotic aromatic compounds are the raw materials of necessities in modern life, such as plastics, pesticides, and antibiotics. To meet the global requirements, their production and consumption have continually increased, and thus, the vast amount of waste generated results in prominent environmental pollution. Fortunately, some microorganisms (e.g., *Comamonas* spp.) can specially use these pollutants as substrates for growth, allowing for the development of bioremediation technology to achieve sustainable development goals. Here, we describe common xenobiotic aromatic compounds used in our daily life, discuss their impact on the environment, and review their biodegradation strategies by *Comamonas testosteroni*, as an example. Finally, we argue that microbiome engineering opens up the avenue to future biofilm-based biodegradation technology to improve aromatic compound bioremediation.

## 1. Introduction

Early in 1825, Faraday presented a paper on “two new compounds of carbon and hydrogen” at the Royal Society and, for the first time, which reported the isolation of the aromatic benzene [1]. This was the first aromatic compound isolated by humans. Since the discovery of the benzene ring structure, researchers have found more and more homologous compounds of benzene. Broadly, we refer to a benzene ring hydrocarbon molecule that contains at least one delocalized bond as an aromatic compound. However, people also find that more and more compounds have aromaticity but do not have a benzene ring structure. Thus, this definition has been expanded to include a broader spectrum of compounds [2].

**Figure 1:**
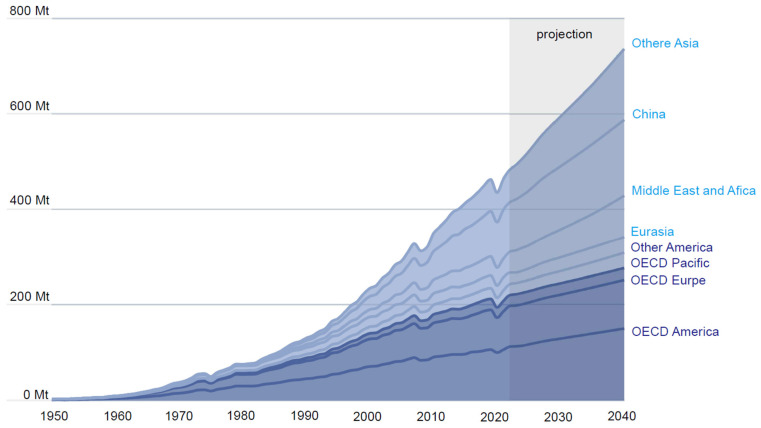
Plastics use projections. Global growth in plastics production and use will continue to outpace population growth. Source: OECD (2024), Policy Scenarios for Eliminating Plastic Pollution by 2040 [12].

The structures and characteristics of aromatic compounds are diverse, and therefore, their properties and uses are different. The strong π-π stacking phenomenon between aromatic molecules provides a noncovalent interaction force for some chemical or biological processes [3]. Also, the nucleophilic substitution of aromatic rings provides a theoretical basis for people to construct new aromatic compounds [4,5]. We refer to these artificial aromatic compounds as xenobiotic aromatic compounds, which have been widely used in pharmaceutical products and daily necessities, such as dyes, drugs, pesticides, herbicides, insecticides, explosives, and other industrial chemicals [6]. In the pharmaceutical industry, synthetic benzenoid alternatives are also essential for producing new small-molecule drugs because aromatic compounds account for 45% of the market [4,7]. Given environmental sustainability, the human health risks arising from aromatic compounds mainly come from using drugs, plastics, pigments, and pesticides [8,9]. For example, global growth in plastics production and use will continue to outpace population growth (Figure 1). Plastics production doubled between 2000 and 2019, from 234 to 460 million tons. Without more targeted policies, the quantity of plastics produced around the world will continue to rise. The global production and use of plastics are set to reach 736 million tons (Mt) by 2040, up 70% from 435 Mt in 2020, as demand for plastics is projected to remain high and further increase in OECD (Organization for Economic Co-operation and Development) countries. Therefore, developing sustainable policies to mitigate the impacts of plastics, antibiotics, and pesticides on the environment is urgently needed [10,11].

Currently, mitigation of the pollution of xenobiotic aromatic compounds can use chemical, physical, or biological technologies [13]. Chemical technologies for removing or degrading aromatic pollutants have been proven more efficient than the other two approaches. However, this treatment is not ecologically friendly because other chemicals are usually added to react with the contaminants. Further, the high cost of chemical use should not be ignored. Physical adsorption or sequestration of aromatic compound pollutants using natural or synthetic materials is a sustainable policy. Nonetheless similar to chemical treatment, this method also has a high cost of materials production, and adding these foreign materials into the local ecosystem may result in secondary pollution. By contrast, biodegradation or bioremediation of pollutants has been highly prioritized, especially when our society calls for the 17 Sustainable Development Goals (SDGs, https://sdgs.un.org/goals (accessed on 20 October 2024).

In this mini-review, we summarize the typical xenobiotic aromatic compounds applied in our society, discuss their impact on the environment, and review the state-of-the-art biodegradation strategies of these pollutants by *Comamonas testosteroni*, an emerging pollutant-degrading bacterium [14]. We argue that gathering research information related to the degradation of specific aromatic compounds can provide assistance for future microbiome engineering in environmental pollution control.

## 2. Environmental Impact of Xenobiotic Aromatic Compounds

### 2.1. Plastics-Related Aromatic Compounds

**Figure 2:**
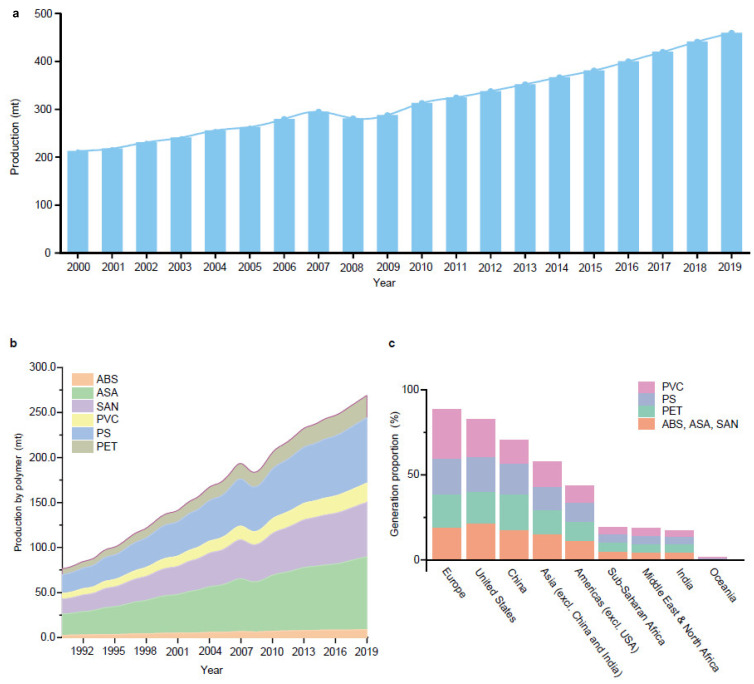
Global production and consumption of plastics. (**a**) Global production of plastics from 1990 to 2019. (**b**) Global production of primary aromatic-related plastics by polymer from 1990 to 2019. (**c**) Aromatic-related plastic waste generation proportion by region and polymer in 2019. Polymer types are as follows: Acrylonitrile Butadiene (ABS), Acrylonitrile Styrene Acrylate (ASA), Styrene Acrylonitrile (SAN), Polyvinyl Chloride (PVC), Polystyrene (PS), and Polyethylene Terephthalate (PET). Source: https://www.oecd.org/ (accessed on 20 October 2024).

Plastics have become indispensable in modern life because of their excellent resistance and insulation, and stable chemical properties [15]. However, the majority of plastics, such as polystyrene (PS), phthalates (plasticizers), polyphenylene sulfide engineering plastics (PPS), polyethylene terephthalate (PET), styrene-acrylonitrile copolymer (SAN), acrylonitrile butadiene styrene (ABS), and bisphenol A polycarbonate (PC), are made from aromatic compounds. Although plastics have similar molecular skeletons to those of aromatic compounds, their physicochemical properties are highly different due to their chemical structures (Table 1). These properties make them the necessities of our society, and thus, their production and consumption have continuously increased year by year (Figure 2a,b).

However, the vast quantity of plastics has increasingly threatened environmental and ecological health all over the world (Figure 2c). Due to their durability, the lifespan of plastics is estimated to be up to hundreds to thousands of years. This property has vastly increased the difficulty of treating plastic pollution in the environment, especially in marine ecosystems [15]. According to one of the latest model surveys, our society’s consumption of plastics is still increasing, indicating that environmental risks arising from the use of plastics will increase [16]. For example, the impact caused by marine microplastics is rather severe. It is reported that the content of microplastics on Guangzhou beach reached 6,701,375 items/m^2^ [17]. Developed countries like the United States also have severe microplastic pollution in their water bodies, comprising 6,698,264 ± 3,929,093 items/km^2^ in the Chicago River [18]. In addition, the Arctic, which is far from human life, is also contaminated by microplastics [15]. The abundance of microplastics present in this environment can cause the accumulation of microplastics in organisms. These organisms cannot degrade the microplastics in the body, ultimately leading to severe impacts on normal life metabolism, affecting organisms’ normal eating and digestive functions [19,20]. More significantly, microplastics entering organisms can trigger inflammatory reactions and reduce the stability of membranes in digestive system cells [21]. Further, certain additives in plastics can also act as endocrine disruptors and affect the growth and development of the human body [20]. For example, plasticizers, like bisphenol A in PC, have been proven to cross the placental barrier and have been shown to influence gene expression during fetal brain development [22]. Thus, developing efficient measures to mitigate the impact of plastics-related aromatic compounds is a global request.

### 2.2. Pesticides-Related Aromatic Compounds

**Figure 3:**
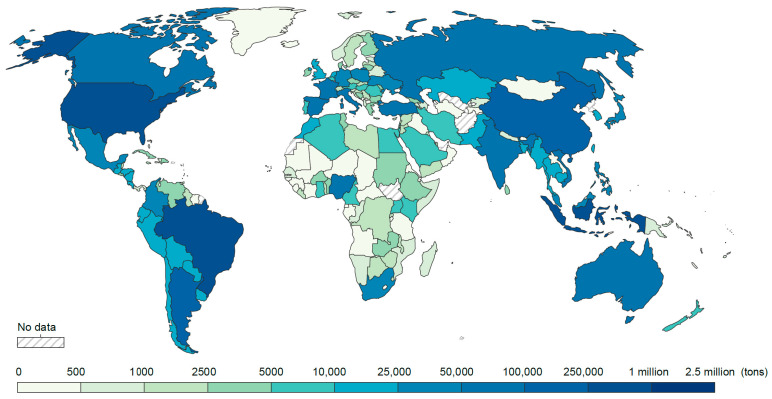
Global consumption of pesticides in 2021. Data were extracted from The FAOSTAT Pesticides Use: https://www.fao.org/faostat/en/#data/RP (accessed on 15 October 2024).

At present, pesticides are used frequently to meet the requirements of modern agriculture. The Food and Agriculture Organization of the United Nations (FAO) estimates that food production will increase by 80% by 2050 to keep up with the growing population. Consequently, pesticides will continue to play a role in agriculture. According to their intended use, pesticides can be roughly divided into insecticides, fungicides, and herbicides. The most commonly used pesticides globally are herbicides, accounting for approximately 47.5% of the total usage; insecticides, accounting for 29.5%; fungicides, accounting for 17.5%; and other pesticides, accounting for 5.5% [23]. The United States, China, Russia, Brazil, and Australia are the countries with the most significant pesticide use in 2021 (Figure 3). Many pesticides are aromatic compounds, such as imidacloprid, cyclofluconazole, and pyrazosulfuron (Table 2). These pesticides are difficult to naturally degrade in the environment after use, and these pesticides are classified as endocrine disruptors [24,25]. Therefore, the environmental pollution caused by pesticides should not be underestimated.

Unlike plastics, pesticides pose a more direct and significant environmental threat due to their inherent toxicity. Pesticides present in the environment can affect various aspects of the ecology. Some studies have shown that when rats are exposed to an environment containing pesticides, the cancer incidence rate significantly increases [24,25]. Taking fipronil as an example, its half-life in the environment is very long, ranging from 28 to 34 days [26]. When people are continuously exposed to this pollutant environment, the secretion of the thyroid-stimulating hormone (TSH) in the human body may be inhibited [27]. When rats are continually exposed to fipronil, it can lead to a significant increase in the TSH concentration of the serum and a substantial increase in the probability of developing thyroid cancer [27,28]. It raises the possibility that fipronil has a central inhibitory effect on TSH secretion in humans. Pyrazosulfuron ethyl is a widely used herbicide, but its potential toxic effects are poorly understood. Recent observations showed that pyrazosulfuron ethyl exposure causes various developmental defects, including reduced survival, shorter body length, and higher deformation rates [29].

### 2.3. Antibiotics-Related Aromatic Compounds

Since the discovery of penicillin, the mortality rate of bacterial infections has dramatically decreased. For instance, the mortality rate due to infectious diseases in the UK was about 25% before the 1900s. However, this rate decreased to less than 1% after the introduction of commercially used antibiotics [30]. Early antibiotics often caused side effects to the human body, so antibiotics have now developed into the fifth generation [31]. As discussed above, substitution in the benzene ring plays an essential role in the production of small-molecule drugs, and it also plays a crucial role in the production of antibiotics [4]. The convenience resulting from the modification of the benzene ring has also promoted the development of antibiotics. Common aromatic antibiotics, such as oxacillin sodium, amoxicillin, cephalexin, and tetracycline, are types of aromatic compounds (Table 3).

**Figure 4:**
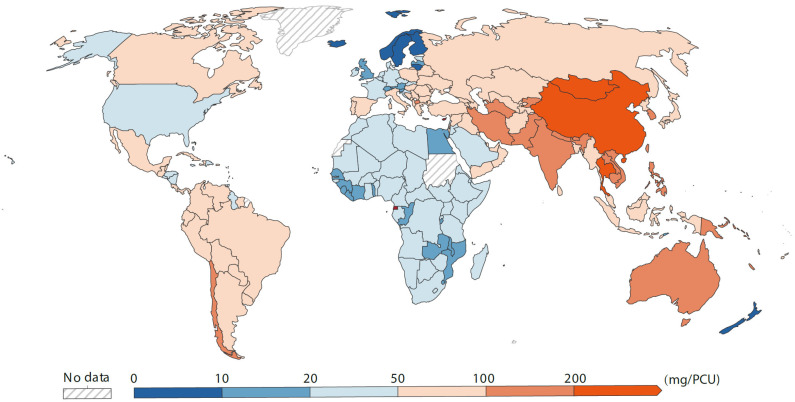
Global consumption of antibiotics for livestock in 2020. Data are shown as the milligrams of antibiotics used per kilogram of livestock, standardized to the Population Correction Unit (PCU) [37]. Adjustments were made for differences in livestock numbers and species.

These antibiotics all have two sides. For example, tetracycline was once considered one of the most toxic antibiotics to ecosystems [32]. With the use and development of antibiotics, the environmental risks arising from antibiotic abuse are becoming increasingly apparent (Figure 4). In addition, antibiotics can alter microbial colony structure and induce the formation of resistance genes in microorganisms [33,34]. Notably, antibiotics entering the environment can allow bacteria in water and soil to develop different antibiotic-resistance genes [33]. For example, most livestock facilities directly discharge wastewater or excrement into the natural environment without treatment, and the level of veterinary antibiotics is often detectable. Therefore, current research interests focus primarily on the environmental hazards of veterinary antibiotics [34,35]. When organisms are exposed to an environment polluted by antibiotics (e.g., tetracycline and sulfonamides), their normal metabolism will be largely disrupted. Studies have shown that many plants could spontaneously absorb, accumulate, and degrade antibiotics. Therefore, some plant organs, like roots, could be damaged once the accumulation of tetracycline and sulfonamides in plants reaches a certain level [35]. Similarly, insects are also affected by residual antibiotics in soil. When earthworms are directly exposed to various concentrations of antibiotics, there is a clear dose–effect relationship; that is, when the concentration of tetracycline in the environment increases to a certain level, the DNA of earthworms will be damaged [36].

## 3. Biodegradation Strategies for Aromatic Compounds in *C. testosteroni*

*Comamonas* spp. are Gram-negative, rod-shaped bacteria ubiquitous in different environments. *Comamonas testosteronei* is named after its ability to degrade steroid substances such as testosterone. Compared to the common aromatic compounds degrading bacteria *Pseudomonas*, it exhibits less pathogenicity in clinical medicine due to fewer reported cases of disease caused by it. Common *Pseudomonas* species can produce toxic metabolites such as pyocyanin, but there have been no similar reports on *C. testosteroni*. It has higher safety in environmental pollution treatment. Because it can degrade both aromatic and steroid compounds, it may have more potential in more structurally similar compounds.

**Figure 5:**
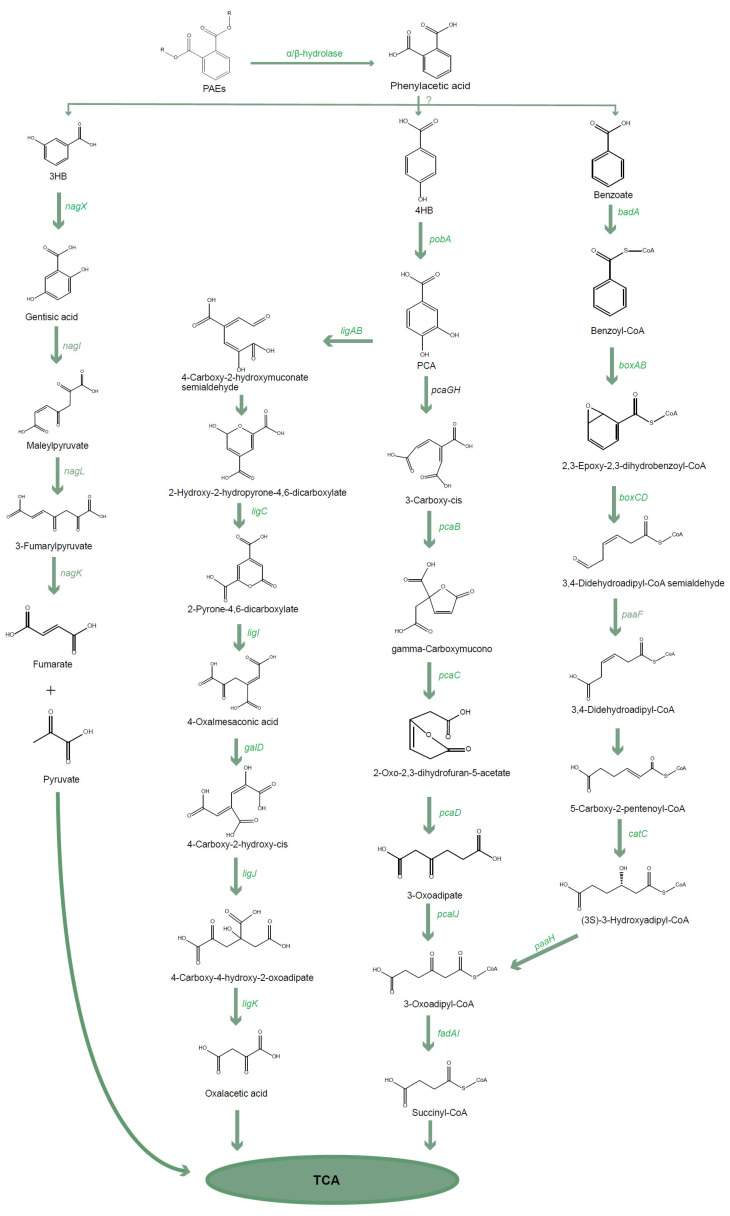
Biodegradation pathway of aromatic compounds in *Comamonas testosteroni*. The pathway includes three benzene ring openings corresponding with the monooxygenase treatment pathway, the dioxygenase treatment pathway, and the monooxygenase pathway under the influence of coenzyme A. The question mark in the figure indicates that the specific reaction process is unknown. PAEs, phthalic acid esters; 4HB, p-Hydroxybenzoic acid; 3HB, 3-Hydroxybenzoic acid.

*C. testosteroni* can degrade complex xenobiotic compounds and has been reported to be a promising microorganism for the bioremediation of recalcitrant organics-contaminated environments [38]. Importantly, microorganisms have been found to develop one to four strategies for the degradation of aromatic compounds [39]. We will focus on *C. testosteroni* to discuss their biodegradation strategies for aromatic compounds (Figure 5).

### 3.1. Biodegradation Pathways in C. testosteroni

In earlier studies, *C. testosteroni* was employed to degrade environmental hormones, such as estrogen [40]. However, recent observations showed that *C. testosteroni* has excellent potential to degrade aromatic compounds [41]. More efforts have been made to explore and elucidate metabolic pathways of *C. testosteroni* in the degradation of aromatic compounds to improve biodegradation. Studies on biodegradation pathways of aromatic compounds indicated that protocatechuic acid (PCA) is one of the essential intermediates in bacteria [38,42]. PCA’s ring opening mode under aerobic conditions can be achieved through *ortho* cleavage at 3,4, or *meta* cleavage at 2,3, and 4,5 [6,39,43]. Notably, the critical enzyme, protocatechuate 4,5-dioxygenase (P4,5D), which catalyzes the 4,5 *meta* cleavage, has been discovered in *C. testosteroni* [44], indicating the biodegradation ability of aromatic compounds (Figure 5).

Typically, *C. testosteroni* could use some single-ring halogen-free aromatic compounds, such as vanillate, 3-hydroxybenzoate (3HB), and 4-hydroxybenzoate (4HB), as carbon and energy sources, by converting them into PCA using oxygenases [38,44,45]. For example, the first step in phthalate degradation is initiated by phthalate dioxygenase (PDO) [45]. However, other studies also indicated that the degradation pathway of PCA in *C. testosteroni* can be achieved only through cleavage at the 4,5 position, but not at the 2,3 and 3,4 positions because of a lack of the corresponding functional genes [14]. Based on the 4,5 cleavage, PCA will be degraded into various intermediates through catalysis by six enzymes [14]. Firstly, PCA is cleaved into 4-carboxy-2-hydroxymyconate-6-semi aldehyde (CHMS). Next, CHMS is transformed into its intramolecular semi-acetal form and oxidized to 2-pyrone-4,6-dicarboxylate (PDC), which is converted into 4-oxalomesaconate (OMA) and then into 4-carboxy-4-hydroxy-2-oxoadipate (CHA). Finally, CHA is converted into oxaloacetate (OAA) and pyruvic acids through aldose cleavage [46]. However, it should be noted that some mechanisms of biodegradation of aromatic compounds are still unknown in *C. testosteroni*. For example, the gene *pmdF* encoding CHA aldolase is responsible for the last step of the PCA 4,5 cleavage into pyruvic acids in *C. testosteroni* CNB-1. Still, this gene is not found in *C. testosteroni* KF-1, indicating the potential existence of a novel biodegradation different from the strain CNB-1 [14,46]. Moreover, the degradation mechanism of *C. testosteroni* at positions 2,3, and 3,4 of PCA is still unclear. Thus, further studies are required to unravel the aromatic compound degradation pathway before reaching the central carbon metabolism (CCM).

In addition to converting the above-mentioned aromatic compounds into PCA, there are other complex degradation pathways in *C. testosteroni*. For example, *C. testosteroni* has been shown to have the ability to degrade biphenyls, but the metabolism is incomplete. Taking the degradation of 2,2′-and 3,3′-dihydroxyphenyl as an example, the final metabolites, such as 2,3,2′-trihydroxyphenyl and 3,4-dihydroxy-5-(3′-hydroxyphenyl)-5-cycloxen-1-one, cannot be further degraded by *C. testosteroni* [47]. Some studies showed that there might be new mechanisms for oxidation of the benzene ring. The enzymes encoded by nine genes (i.e., *cnbA*, *cnbB*, *cnbCa*, *cnbCb*, *cnbD*, *cnbE*, *cnbF*, *cnbG*, and *cnbH*) have been confirmed in *C. testosteroni* CNB-1, which are responsible for further biodegradation pathways from 4-chloronitrobenzene to 5-chloro-4-hydroxy-2-oxovalenoic acid [48]. Other observations demonstrated the degradation of hexachlorobenzene in *C. testosteroni.* The authors proposed a hypothesis on the degradation pathway of hexachlorobenzene: hexachlorobenzene is first degraded by replacing the chlorine atoms at position 1,6 with hydroxyl groups, then dechlorinated at position 4,5, and finally added with an oxygen atom to break the benzene ring at position 2,3 [49]. After the above degradation processes, the final products are succinyl-CoA and acetyl-CoA, which then enter the tricarboxylic acid cycle.

Another CoA-dependent epoxide biodegradation strategy could be employed by *C. testosteroni* CNB-1 when using benzoate as the substrate. The CoA-dependent epoxide pathway begins with the transformation of benzoate to benzoyl CoA by the benzoate CoA ligase. Benzoyl CoA is then converted into an epoxide (2,3-epoxybenzoyl CoA) by benzoyl CoA oxygenase (BoxB) and reductase (BoxA), and the latter is hydrolyzed into formic acid and 3,4-dehydrohexacyl CoA semialdehyde by benzoyl CoA hydratase (BoxC). Under the catalysis of 3,4-dihydroadipate CoA semialdehyde dehydrogenase (BoxD), the semialdehyde is subsequently oxidized to 3,4-dehydroadipyl-CoA. Further biodegradation of 3,4-dehydroadipyl-CoA is similar to the β-oxidation and β-ketoadipate pathways with succinyl-CoA and acetyl-CoA as final products [50].

However, all such biodegradation pathways lack evidence from chemical experiments [14]. Further work through chemical biology methods is still required to confirm the biodegradation strategies.

### 3.2. Key Functional Genes and Enzymes

In *C. testosteroni*, the functional genes and enzymes responsible for various biodegradation strategies are diverse, even for the same aromatic compound. For example, phthalates are not only a common plastic plasticizer but also the degradation intermediates of PAEs and polycyclic aromatic hydrocarbons (PCBs) [51]. Many observations showed that phthalate biodegradation is initiated by phthalate dioxygenase (PDO) and reductase (PDR), which are encoded by the genes *phtA* and *phtB*, respectively [45]. The gene clusters related to phenol degradation are *aphCEFGHJI* and *aphKLMNOPQB*, but these two gene clusters are only found in some *C. testosteroni* strains [52]. Moreover, nitrobenzene biodegradation is determined by the gene cluster *cnb*, as discussed above [48]. Currently, the degradation pathway via cleavage at position 4,5 is the most fully explained for PCA biodegradation. This pathway is sequentially activated by the following six enzymes: protocatechuate 4,5-dioxygenase α-subunit, protocatechuate 4,5-dioxygenase β-subunit, 4-carboxy-2-hydroxymuconate-6-semialdehyde dehydrogenase, PDC hydrolase, 4-oxalomesaconate tautomerase, 4-oxalomesaconate hydratase, and 4-carboxy-4-hydroxy-2-oxoadipate aldolase/oxaloacetate decarboxylase, which are controlled by the *pmd* operon [46]. Importantly, the operon *pmd* has been widely discovered in *C. testosteroni* strains, and the potential to degrade various heterologous aromatic compounds has also been confirmed at the gene level [53].

Although many functional genes responsible for aromatic compound degradation have been discovered, a vast gene pool in *C. testosteroni* needs to be explored to unravel novel biodegradation pathways.

### 3.3. Regulatory Mechanisms

To date, very few regulatory mechanisms have been discovered for aromatic compound degradation in *C. testosteroni*. One study on the degradation ability of *C. testosteroni* to phenol found that *C. testosteroni* TA441 initially cannot grow on phenol, but this ability is subsequently exhibited after a period of exposure to a phenol-containing environment [52]. This indicates that aromatic compounds present in the environment have an inducing effect on gene expression for phenol degradation. Moreover, recent studies on the 4,5 position degradation of PCA found that growth on aromatic substrates triggers upregulation of the 4,5 pathway transcripts, transcription regulatory factors, and proteins relative to succinate growth by more than 18 times. Also, the corresponding enzymes involved in the transportation and oxidation of aromatic compounds were detected or differentially expressed when growing on different substrates. The growth on aromatic substrates triggers the upregulation of gene products related to substrate transport and initial catabolism towards the 4,5 pathways. Importantly, OAA in the 4,5 pathways can serve as the enzyme inhibitor to regulate the carbon flux entering CCM, which further influences the TCA cycle fluxes.

Microbial degradation of aromatic compounds is more diverse than scientists had thought. The classical strategy of O_2_-dependent ring cleavage is just one strategy to overcome the aromatic ring’s high chemical stability. There is an overwhelming variety of mechanisms and chemical reactions behind these biodegradation strategies, some of which have only rare counterparts in other branches of organic chemistry. Thus, more discoveries of unknown biodegradation strategies could be achieved with the help of both biologists and chemists.

## 4. Concluding Remarks

Due to global consumption growth, the environmental impact of xenobiotic aromatic compounds will continuously increase in the following decades. Although developing green and sustainable alternatives for xenobiotic aromatic compounds is primarily encouraged, the current production of alternatives is far from the requirements of our society. Microbial biodegradation is the best choice for the sustainable treatment of such pollutants, enabling its wide application in soil remediation, sewage treatment, and solid waste degradation [41,54,55]. However, the limited chemical reactivity of aromatic compounds makes them difficult to degrade in the environment. Thus, understanding the biodegradation mechanisms of environmental microorganisms will help advance bioremediation technologies in the contaminated environment.

The most challenging problems for future studies concern unravelling the reaction mechanisms of biodegradation step by step. Unfortunately, the majority of enzymes involved in biodegradation are unclear, and we lack efficient measures for accurately identifying the degraded product. Despite this, modern multi-omics could give us more information on both individuals and microbiomes that thrive in the environment contaminated by the corresponding aromatic compound [56]. Guided by this valuable information, researchers can adopt a bottom-to-top strategy to selectively construct a microbiome that improves pollutant degradation [57]. For example, researchers may choose *C. testosteroni* as the starter because it can initiate the biodegradation of aromatic compounds and then selectively couple it with other community members that grow on its degraded intermediates. Using this strategy, researchers may develop a multispecies biofilm-based biodegradation model for aromatic compounds, and can further optimize this model by evaluating the biodegradation efficiency before practical application.

## Figures and Tables

**Table 1:** Several plastics-related aromatic compounds.

Name	Structure	Main Function
Polyethylene terephthalate	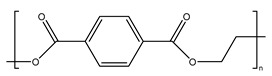	It is mainly used for electrical components and plastic bottles, most of which are recyclable.
Polyphenylene sulfide	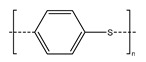	A high-quality, efficient, and high-temperature-resistant material.
Polystyrene	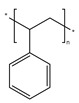	It is often used to make foam plastic products.
Bisphenol-A-polycarbonate (PC)	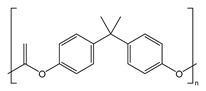	An engineering plastic used for making transparent components
Polyimide	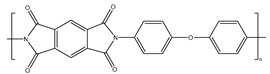	Used as a high-temperature insulation material

**Table 2:** Several pesticide-related aromatic compounds.

Type	Name	Structure	Main Function
Insecticide	Carbaryl	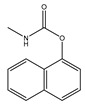	Prevention and control of cotton bollworms, leaf rollers, etc.
	Fipronil	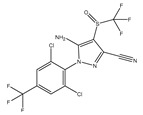	Used for preventing and killing cockroaches, ants, etc.
	Teflubenzuron	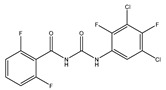	Inhibition of chitin synthesis
Herbicide	Bipyrazone	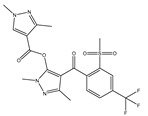	HPPD inhibitor herbicides for broad-leaved grass in wheat fields
	Pyrazosulfuron-ethyl	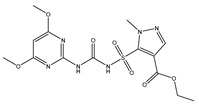	Selective systemic conductive herbicide
	Cypyrafluone	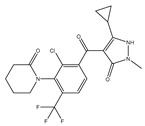	HPPD inhibitor herbicides for wheat field grasses in the Poaceae family
Germicide	Thiophanate-Methyl	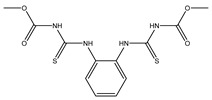	Broad spectrum systemic low-toxicity fungicide
	Carbendazim	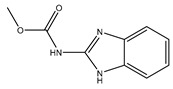	Effective prevention and control of various crop diseases caused by fungi
	Fenaminosulf	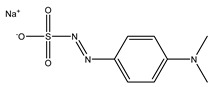	Protective fungicides used for the prevention and control of vegetable diseases

**Table 3:** Structure and usage of some common aromatic antibiotics.

Name	Structure	Main Function
Amoxicillin	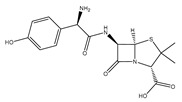	Used for penicillin-resistant *Staphylococcus aureus* infection
Oxacillin sodium salt	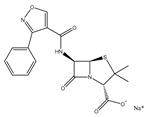	Broad-spectrum antibiotics suitable for infection caused by sensitive organisms (strains that do not produce β-lactamase)
Cephalexin	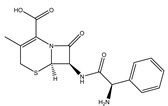	Broad-spectrum antibiotics suitable for respiratory infections caused by sensitive organisms
Tetracycline	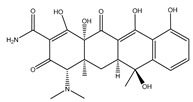	They are widely used in infections caused by Gram-positive and harmful bacteria, intracellular Mycoplasma, Chlamydia, and Rickettsia.
